# 2‐Deoxyglucose and hydroxychloroquine HPLC‐MS–MS analytical methods and pharmacokinetic interactions after oral co‐administration in male rats

**DOI:** 10.1002/prp2.1173

**Published:** 2024-01-31

**Authors:** Dongxiao Sun, Sangyub Kim, Deepkamal Karelia, Yibin Deng, Cheng Jiang, Junxuan Lü

**Affiliations:** ^1^ Department of Pharmacology Pennsylvania State University College of Medicine Hershey Pennsylvania USA; ^2^ Pennsylvania State University College of Medicine Mass Spectrometry Core Facility Hershey Pennsylvania USA; ^3^ Department of Urology University of Minnesota College of Medicine Minneapolis Minnesota USA; ^4^ Penn State Cancer Institute Hershey Pennsylvania USA

**Keywords:** 2‐deoxyglucose, drug–drug interaction, hydroxychloroquine, oral drug–drug PK interference

## Abstract

Our previous work has shown a synergistic tumoricidal efficacy of combining the hexokinase (HK) inhibitor 2‐deoxyglucose (2‐DG) and the autophagy inhibitor chloroquine (CQ) through intraperitoneal injections on HK2‐addicted prostate cancers in animal models. The pharmacokinetic (PK) behaviors of these oral drugs after simultaneous oral administration have not been reported. We developed high‐performance liquid chromatography–tandem mass spectrometry (HPLC‐MS–MS) analytical methods for 2‐DG and the clinically favored drug hydroxychloroquine (HCQ) for sera samples. Using a jugular vein‐cannulated male rat model with serial blood collection before and after a single gavage dose of each drug alone or in combination, we examined their PK metrics for drug–drug interactions. The data demonstrated a rapid and complete separation of 2‐DG from common monosaccharides by HPLC‐MS–MS multi‐reaction monitoring. Application of the HPLC‐MS–MS 2‐DG and HCQ methods to sera samples of nine rats showed a peak time (*T*_max_) for 2‐DG of 0.5 h after 2‐DG alone or with HCQ and identical post‐peak half‐life of approximately 1 h. With a seemingly bi‐modal time course for HCQ, the *T*_max_ for HCQ alone (1.2 h) was faster than that for the combination (2 h; *p* = .017). After combination dosing, the peak concentration (*C*_max_) and area under the curve (AUC_0‐4h_) of 2‐DG were decreased by 53.8% (*p* = .0004) and 53.7% (*p* = .0001), whereas AUC_0‐8h_ for HCQ was decreased by 30.8% (*p* = .0279) from the respective single dosing. Without changing the mean residence time (MRT_0‐∞_) of each drug, the combination affected the apparent volume of distribution (*V*_d_) and clearance (CL) of 2‐DG, and CL for HCQ without affecting its *V*_d_. We observed significant negative PK interactions, probably at the intestinal absorption level, between 2‐DG and HCQ taken simultaneously by mouth. Future optimization efforts are warranted for their combination regimen for clinical translation.

## Graphical abstract

Serum concentration versus time curves after single dose alone or combination dosing. (A) 2‐DG; (B) HCQ.

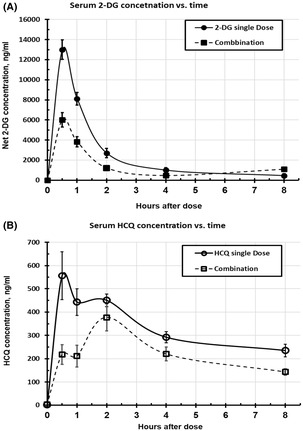

**Abbreviations**
(2‐DG): 2‐deoxyglucose
AMPK: AMP‐activated protein kinase
AUC: area under the curve
CL: clearance
*C*_max_
: peak concentration
CQ: chloroquine
DDI: drug–drug interaction
GC: gas chromatography
HK: hexokinase
HPLC: high performance liquid chromatography
IS: internal standard
LLOD: lower limit of detection
LLOQ: lower limit of quantitation
MRM: multi‐reaction monitoring
MRT_0–∞_
: mean residence time
MS–MS: tandem mass spectrometry
PK: pharmacokinetic
RI: radioisotopic
*t*_1/2_
: half‐life
*T*_max_
: peak time
UV: ultraviolet detection
*V*_d_
: volume of distribution

## 1 INTRODUCTION

2‐Deoxyglucose (2‐DG) https://www.guidetopharmacology.org/GRAC/LigandDisplayForward?ligandId=4643 is a synthetic glucose analog in which the 2‐hydroxyl group is replaced by a hydrogen atom. 2‐DG, like glucose, enters the glucose‐craving cancer cells through glucose transporters and is phosphorylated by a hexokinase (HK) https://www.guidetopharmacology.org/GRAC/FamilyDisplayForward?familyId=890, isoform(s), the first and rate‐limiting enzyme in glycolysis. Not further metabolizable, the HK product 2‐DG‐6‐phosphate accumulates in cells and competitively inhibits HK and glycolysis, leading to cancer cell cycle arrest and autophagy, the latter promoting cancer cell survival by antagonizing apoptosis.^1^ Due to its cytostatic nature, 2‐DG monotherapy had little efficacy in early‐stage human clinical trials for the treatment of cancer‐including prostate cancer.^2^

For decades, chloroquine (CQ) https://www.guidetopharmacology.org/GRAC/LigandDisplayForward?ligandId=5535 and hydroxychloroquine (HCQ) https://www.guidetopharmacology.org/GRAC/LigandDisplayForward?ligandId=7198 have been orally available drugs for preventing and treating malaria.^3^ Known as lysosomotropic autophagy inhibitors, they are also indicated for treating and managing autoimmune diseases such as lupus and rheumatoid arthritis.^4^ Based on the rationale that an induction of autophagy in HK2‐overexpressing and addicted prostate cancer cells by 2‐DG counteracts their cell death by apoptosis, our previous work has shown a synergistic tumoricidal action of the 2‐DG and CQ combination, through intraperitoneal injections, in several prostate cancer animal models.^1^ Because 2‐DG exposure of prostate cancer cells in vitro took between 4 and 6 h to activate AMP‐activated protein kinase (AMPK) to drive the autophagy induction, the daily dosing regimen (Monday–Friday) in the tumor‐bearing mice was based on such a signaling consideration and carried out with 2‐DG injection in the morning and CQ injection in the afternoon in our previous work.^1^

Pharmacokinetic (PK) interaction is one of the main drug–drug interactions (DDIs) and a major cause of medication error.^5^ Since 2‐DG and HCQ are taken by human patients orally, knowledge of their DDIs will inform the optimal dosing regimen to achieve cancer therapeutic benefit over harm. Whereas the PK behavior of each drug has been extensively documented in rodents and humans, their PK interactions, if any, after simultaneous oral administration have not been reported.

Methodologically, different analytical protocols have been reported for 2‐DG previously. One early method used gas chromatography (GC) but required derivatization using N‐trimethylsilylimidazole in pyridine to make it volatile.^6^ Another method analyzed the presence of tritiated ^3^H‐2‐DG in rat muscle using chromatography‐radioisotopic (RI) detection.^7^ Yet another used high‐performance liquid chromatography (HPLC)‐ultraviolet detection (UV) at 195 nm,^8^ due to the lack of a chromophore absorbing above 200 nm in 2‐DG. The analytical columns included a μBondapak 10 μm NH_2_ column and a Varian Micropak 10 μm NH_2_ column. As 2‐DG has a very short retention time on these columns, plus the low sensitivity and selectivity of UV detection, the HPLC‐UV method performed poorly at separating 2‐DG from glucose and other monosaccharides in blood samples. A fluorescence detection method enhanced the sensitivity for 2‐DG analysis through derivatization with 2‐aminobenzoic acid in the presence of sodium cyanoborohydride at 80°C for 45 min.^9^ The subsequent separation by HPLC and detection by fluorescence took an additional 1 h for each sample. The drawbacks, however, included the extra reaction steps, the long HPLC time per sample and not directly measuring the actual analyte.^9^ In summary, no direct method with high sensitivity, selectivity and operational efficiency has been reported for 2‐DG quantitation in biological fluid samples.

In contrast, HPLC with tandem mass spectrometry (HPLC‐MS–MS) has been used for detection of CQ‐family drugs, including HCQ and its major metabolites.^10^, ^11^ HPLC‐MS–MS is a powerful analytical technique that combines the separating power of HPLC with the highly sensitive and selective mass analysis capability of triple quadrupole MS.

Herein, we developed a new HPLC‐MS–MS method for the separation and quantification of 2‐DG in rat sera and modified the HPLC‐MS–MS parameters for the detection of HCQ based on our instrument system. We applied these analytical protocols for 2‐DG and HCQ PK interactions in a jugular vein‐cannulated rat model, which afforded serial PK blood collections following the gavage administration of each drug alone or their combination.

## 2 MATERIALS AND METHODS

### 2.1 Materials

Both 2‐DG (catalog number D6134) and ^13^C_1_‐2‐DG (catalog number 731978) which was used as an internal standard (IS) in initial method development were purchased from Sigma‐Aldrich (St. Louis, MO). ^13^C_6_‐2‐DG was purchased from Toronto Research Chemicals (Toronto, ON, Canada) as the final IS for 2‐DG. HCQ was purchased from TCI Chemicals (Portland, OR, USA) (catalog H1306). Deuterated HCQ‐d4 (HCQ‐d4) was purchased from Toronto Research Chemicals as the HCQ IS. Formic acid was purchased from J.T. Baker (Phillipsburg, New Jersey, USA). Optima LC–MS grade water, acetonitrile and methanol and other chemicals were purchased from Fisher Scientific (Fair Lawn, New Jersey, USA).

### 2.2 HPLC‐MS–MS analysis method for 2‐DG

A new method was developed using a Sciex QTRAP 6500+ MS coupled with an ExionLC separation system (Waltham, MA, USA) and was able to separate 2‐DG from other simple sugars including d‐glucose, fructose, mannose, and galactose (Figure A1). A Luna 3 μm NH2 HPLC column (2 × 100 mm, Phenomenex, Torrance, CA, USA) was used. The isocratic elution was carried out using a flow rate of 0.5 mL/min with water as mobile phase A (17%) and acetonitrile as mobile phase B (83%). The column was kept at 30°C during the separation procedure. The instrument settings and performance metrics are described in detail in Figure A4. The current method's lower limit of quantitation (LLOQ) and lower limit of detection (LLOD) were 63 and 200 times lower than the fluorescence method of Gounder and co‐workers.^9^

### 2.3 HPLC‐MS–MS analysis method for HCQ

An EXionLC separation system with a 1.7 μm Acquity UPLC BEH C18 analytical column (2.1 × 50 mm, Waters, Dublin, Ireland) was used to separate HCQ from other serum constituents. Gradient elution was conducted using a flow rate of 0.3 mL/min with the following conditions: initiate at 2% mobile phase B (acetonitrile) and 98% mobile phase A (0.1% formic acid in water), and linear gradient to 98% mobile phase B in 2 min and keep the mobile phase B at 98% for another 2 min to flush the column before back to the initial conditions to equilibrate the column. The instrument settings and performance metrics are described in detail in Figure A5. The LLOQ for HCQ was 10 times improved over two recently reported LC–MS/MS methods.^10^, ^11^

### 2.4 PK experiments

The animal work was conducted with the approval of the Institutional Animal Care and Use Committee of Penn State College of Medicine, Hershey, PA campus. Jugular vein‐cannulated CD male rats (200–300 g, age 7–9 weeks) were purchased from Charles River, Wilmington, MA. Male rats were used because the 2‐DG‐CQ combination therapy was developed for HK‐2‐addicted prostate cancer models^1^ and intended for precision oncology translation into male patients with a targetable cancer metabolo‐phenotype. The rats were housed individually to prevent damage to the catheter and were provided free access to water and rodent chow pellets. After quarantine and acclimation for 1 week, the rats were used in the PK dosing sequence as shown in Table A1. The number of rats (*n* = 10) was chosen to approximate the number of human PK study subjects (12 or more) as stipulated by FDA. The washout time of 1 day after 2‐DG dosing and 3 days after HCQ dosing was chosen in consideration of the much shorter elimination half‐life for 2‐DG than HCQ. The 2‐DG was dissolved in sterilized saline and gavage‐administered at 372 mg/kg body weight in a volume of 5 mL/kg. HCQ was dissolved in sterilized saline and gavaged at 124 mg/kg body weight in a volume of 5 mL/kg. These doses were based on our early mouse efficacy studies^1^ with inter‐species allometric dose conversion adjustment. The combination dosing was delivered sequentially within 2 min of each other.

On the day of experiment, approximately 0.3 mL of baseline blood was collected into a tube without anti‐coagulant immediately prior to drug dosing, which started the PK clock as 0 h. Sequential blood collections (~0.3 mL each) were performed at the indicated time points ± 5 min (Table A1) followed by proper washout periods between drugs. The PK time points were based on prior knowledge of PK behaviors of each drug^3^, ^9^, ^11^ and our focus on early PK interactions. Serum samples were harvested by centrifugation (1000×*g*, 4°C) and were stored at −80°C for later analyses by HPLC‐MS–MS‐multiple reaction monitoring (MRM) as detailed above. The serum sample preparation procedures for 2‐DG and HCQ measurement are described in detail, respectively, in Figures A6 and A7.

### 2.5 Data presentation and statistical analyses

Nine independent sets of complete data were collected (*n* = 9 rats). For graphical visualization, timepoint group mean and SEM were plotted against blood collection time. Peak time *T*_max_ and peak drug concentration *C*_max_ were determined based on individual rat data and compared between single dosing versus combination dosing by appropriate *t*‐tests (paired or unpaired). The Microsoft Excel add‐in program PKsolver software^12^ was used for non‐compartmental analyses of the individual rat data for PK metrics including post‐peak half‐life *t*_1/2_, mean residence time (MRT_0‐∞_), area under the curve to time and to infinity (AUC_0‐t_ and AUC_0‐∞_), apparent volume of distribution (*V*_d_) and clearance (CL), etc.

### 2.6 Nomenclature of targets and ligands

Key protein targets and ligands in this article are hyperlinked to corresponding entries in http://www.guidetopharmacology.org, the common portal for data from the IUPHAR/BPS Guide to PHARMACOLOGY^13^ (Harding et al., 2018), and are permanently archived in the Concise Guide to PHARMACOLOGY 2019/20^14^ (Alexander et al., 2019).

## 3 RESULTS

### 3.1 Novel HPLC‐MS/MS analysis method for 2‐DG

**FIGURE 1:**
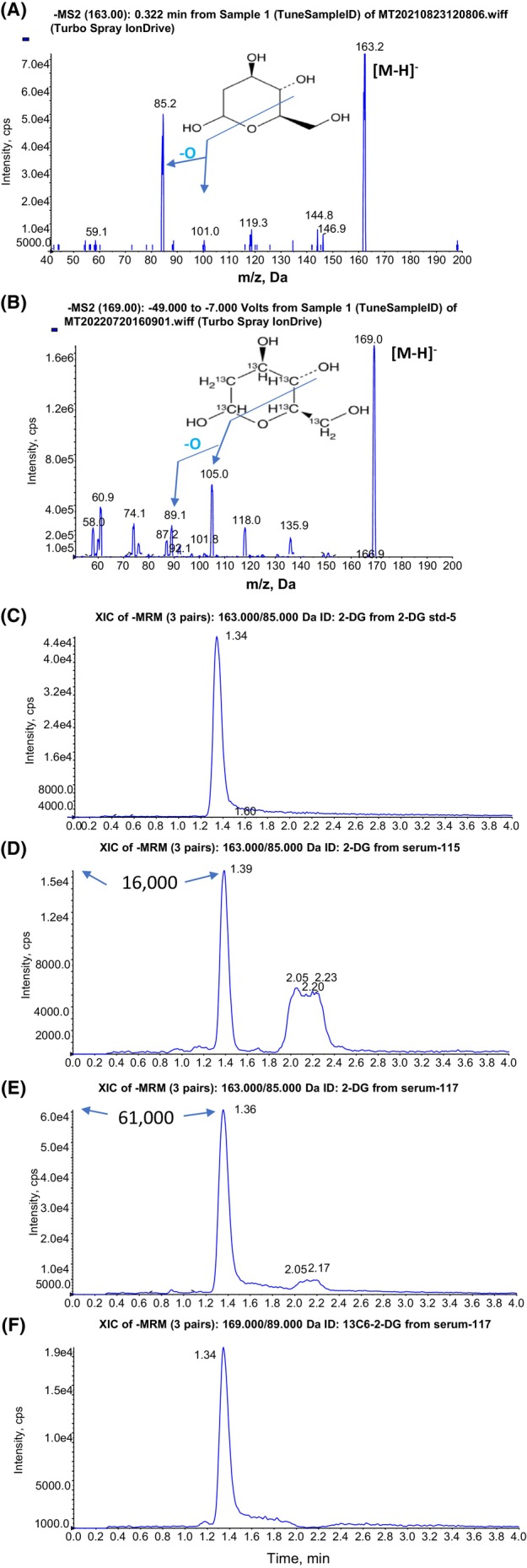
Mass spectra of (A) 2‐DG standard and (B) ^13^C_6_‐2‐DG internal standard (IS) and chromatograms for standard/IS and serum samples (C–F). Product ion scans for A. 2‐DG standard and B. ^13^C6‐2‐DG as internal standard (IS). Fragmentation schema for the product ions for MRM were marked. HPLC‐elution MRM profiles of C. 2‐DG standard; (D) 2‐DG in pre‐dose (control) serum; (E) 2‐DG in post‐dose (1 h treated) serum; (F) ^13^C6‐2‐DG (IS) in post‐dose (1 h treated) serum.

For MS, negative mode was applied for 2‐DG and its IS, ^13^C_6_‐2‐DG. By infusion under Q1 scan analysis, [M‐H]^−^ was found at *m/z* 163 for 2‐DG (Figure 1A) and *m/z* 169 for IS ^13^C_6_‐2‐DG (Figure 1B). By product ion scan, a major fragment of 2‐DG was found at *m/z* 85 (Figure 1A). The m/z 163 → 85 transition was therefore used for MRM. For the IS ^13^C_6_‐2‐DG, fragments at *m/z* 105 and *m/z* 89 were found (Figure 1B). As *m/z* 169 > 89 showed a higher intensity MRM peak than that of *m/z* 169 > 105, we therefore selected the *m/z* 169 → 89 transition for MRM of the IS.

Because of structural similarities with many simple sugars such as glucose, fructose, mannose, galactose, etc. the separation of 2‐DG from these sugars, especially the highly abundant glucose, in serum has always been difficult. We chose a Luna 3 μ NH_2_ HPLC column (2.0 × 100 mm) for method development. The normal phase column was flushed by isopropanol for hours to change it to a reverse phase column for more mobile phase selections and better reproducibility. After careful optimization, an isocratic program of water: acetonitrile (17:83) was used to successfully separate 2‐DG from other monosaccharides (Figure A1). MRM profiles of 2‐DG standard (Figure 1C) and ^13^C_6_‐2‐DG IS (Figure 1F) showed each as a single peak over baseline.

Unexpectedly, we detected an endogenous “2‐DG” peak that eluted at same retention time as that of 2‐DG standard (1.39 min) with identical molecular ion and fragment (*m/z* 163/85) in pre‐dose (control) serum (serum‐115) (Figure 1D). This peak was separated from the other sugars (retention time 2–2.4 min). The chromatograph pattern of 1 h post‐dose serum sample from the same rat dosed with 2‐DG (serum‐117) (Figure 1E) showed sharply increased intensity at the 2‐DG peak, but not at the other sugar peaks (also see Figure A2). This pattern was recapitulated by spiking 2‐DG into the pre‐dose control serum (Figure A3). Since the rats ate laboratory rodent chow pellets made of natural feed ingredients such as wheat, corn, wheat midds, corn gluten meal or soybean meal, we speculate that the endogenous “2‐DG” peak in the pre‐dosing control serum could be an isomer(s) of 2‐DG, possibly a deoxy sugar. Natural deoxy sugars include 6‐deoxy‐l‐galactose, a constituent of cell membrane glycoproteins and glycolipids; 6‐deoxy‐l‐mannose, which resides in plant glycosides; 6‐deoxy‐d‐glucose, a natural product found in *Pogostemon cablin*, *Salmonella enterica*, and other organisms (https://lotus.nprod.net/). As shown later, the endogenous “2‐DG” signal remained stable across PK experiments and was not a problem for assessing 2‐DG PK metrics by using the net 2‐DG values for each time point after subtracting the baseline value.

### 3.2 HPLC‐MS/MS analysis method for HCQ

**FIGURE 2:**
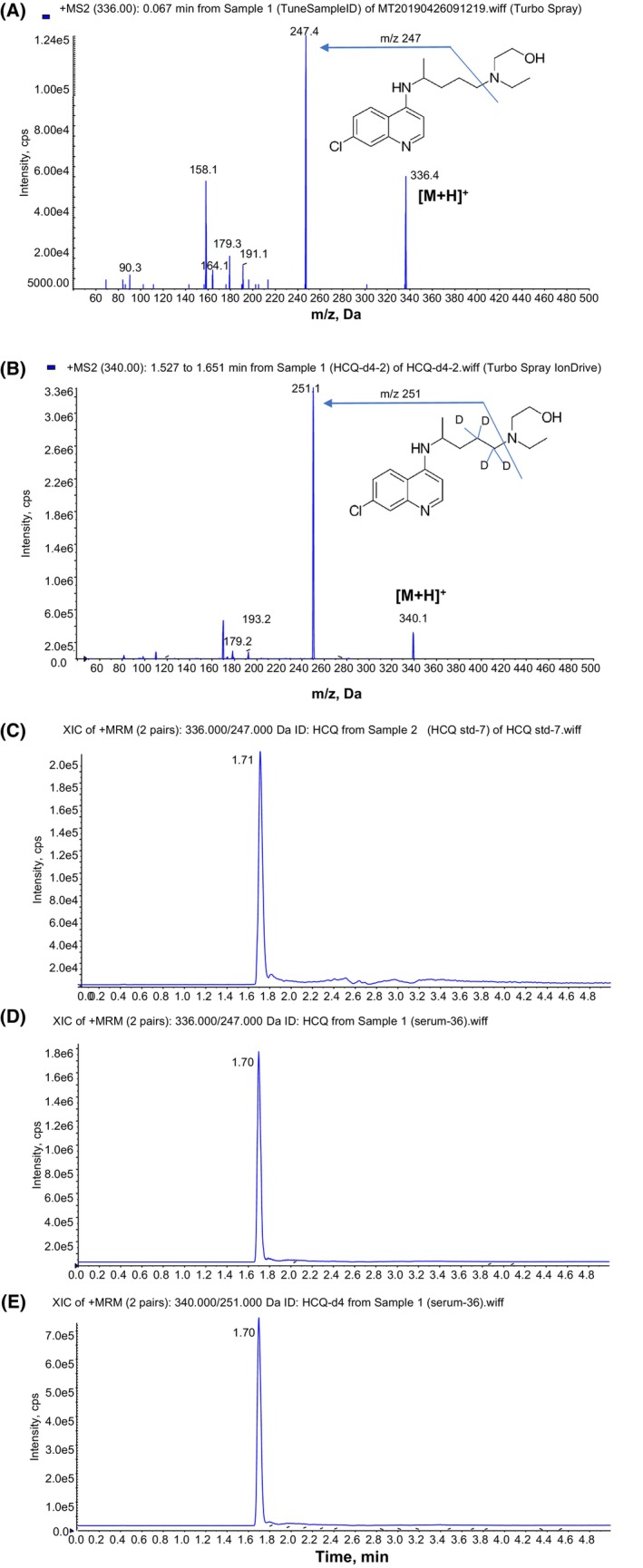
Mass spectra of (A) HCQ standard and (B) HCQ‐d4 internal standard (IS) and chromatograms for standard/IS and serum samples (C–E). Product ion scan for A. HCQ standard and B. HCQ‐d4 as internal standard (IS); HPLC‐elution MRM profiles of C. HCQ standard; (D) HCQ in post‐dose (treated) serum; (E) HCQ‐d4 IS in post‐dose (treated) serum.

To optimize MS conditions, ESI source in positive mode was applied and [M + H]^+^ was found at *m/z* 336 for HCQ (Figure 2A) and *m/z* 340 for HCQ‐d4 as IS (Figure 2B) under Q1 scan analysis. MS/MS product ion scan for the fragmentation of the molecular ions detected specific product ion at *m/z* 247 for HCQ (Figure 2A) and *m/z* 251 for HCQ‐d4 (Figure 2B). Thus, MRM transition of *m/z* 336 → 247 was selected for quantification of HCQ while *m/z* 340 → 251 was selected for quantification of IS HCQ‐d4.

The chromatography conditions were optimized for HCQ analysis with a 5‐min gradient HPLC program using a 1.7 μm C18 column. Sharp peaks with clear baseline were achieved for HCQ standards (Figure 2C) and HCQ‐d4 IS (Figure 2E). When different organic solvents were compared for the extraction of HCQ from serum, methanol plus 0.1% formic acid was found to be the most efficient for a high recovery. Similar patterns were observed for HCQ and HCQ‐d4 peaks in serum sample after extraction (Figure 2D,E). The analytical method developed was specific for the analysis of HCQ in serum, showing no endogenous interfering components at the retention time of the analyte.

### 3.3 2‐DG PK metrics

**FIGURE 3:**
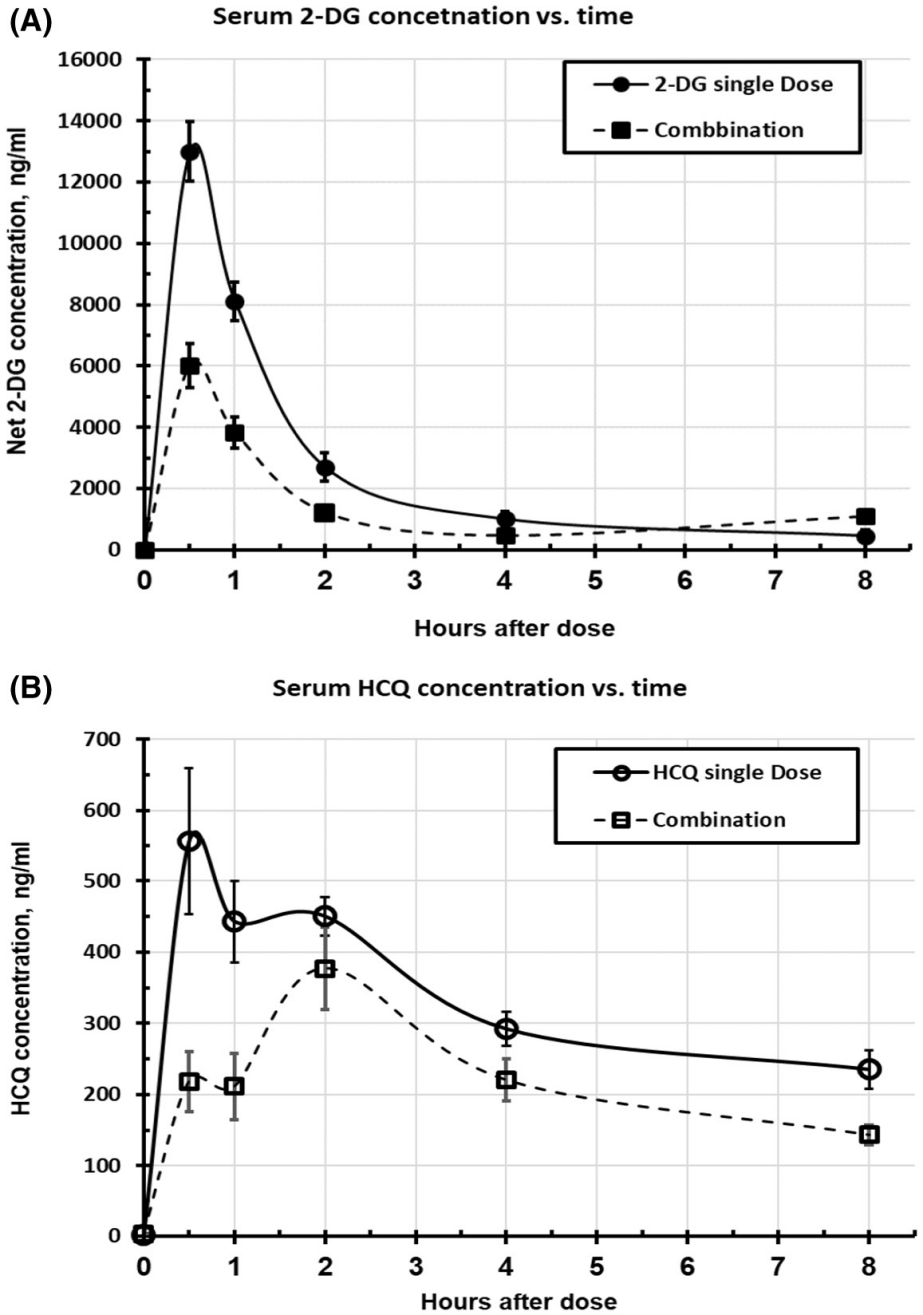
Serum concentration–time curves of (A) 2‐DG and (B) HCQ after respective single dose alone or by combination dosing. Mean ± SEM, *n* = 9.

The endogenous “2‐DG” peak was stable in pre‐dose sera, with no statistical difference between 2‐DG single dose (902 ± 134 ng/mL) and the combination dose (758 ± 66 ng/mL) (2‐tail *t*‐test, *p* = .359). The net 2‐DG concentrations for each rat were therefore obtained by subtracting the corresponding pre‐dose “2‐DG” value. The population PK curve was plotted as the timepoint mean of the group. When dosed alone, 2‐DG (Figure 3A, solid circles, solid line) was taken up rapidly and peaked at 0.5 h (earliest sampled time point) and returned to pre‐dose level by 8 h. However, when 2‐DG and HCQ were dosed together (Figure 3A, solid squares, dash line), the *C*_max_ for 2‐DG was decreased by 53.8% (*p* = .0004) and the AUC_0‐4h_ was reduced by 54.3% (*p* = .0001) (Table 1). Nevertheless, there was no change of the *T*_max_ (0.5 h), the post‐peak half‐life *t*_1/2_ (0.94 vs. 1.04 h) or MRT_0‐∞_ (Table 1). By non‐compartmental analyses, both the apparent volume of distribution (*V*_d_) and clearance (CL) metrics for 2‐DG were significantly increased by the combination dosing with HCQ (Table 1).

**TABLE 1:** Rat PK metrics of 2‐DG and HCQ each given as single dose or a combination dose.

	2‐DG single dosing		Combination dosing			HCQ Single dosing		Combination dosing		
PK	*n* = 9		*n* = 9		Paired	*n* = 9		*n* = 8^a^		Paired
Parameter	Mean	SD	Mean	SD	*t*‐test	Mean	SD	Mean	SD	*t*‐test
*C*_max_ (ng/mL)	13 001	2931	6006	2137	0.0004	627	252	394	178	0.0764
*T*_max_ (h)	0.5	0	0.5	0		1.2	0.8	2.0	0	0.0166
*t*_1/2_ (h)	0.94	0.41	1.04	0.37	0.317	5.90	1.55	5.31	3.72	0.413
MRT_0‐∞_ (h)	1.58	0.56	1.70	0.59	0.357	9.13	2.30	8.49	5.64	0.447
*V*_d_ (L/kg)	0.026	0.011	0.069	0.037	0.009	0.232	0.057	0.347	0.207	0.103
CL (L/h/kg)	0.020	0.005	0.044	0.010	0.00005	0.029	0.009	0.047	0.013	0.0024
AUC_0‐t_ (ng/mL*h)	17 623	3924	8160	2554	0.0001	2632	558	1822	713	0.0279
AUC_0‐∞_ (ng/mL*h)	19 380	4760	8943	2195	0.0001	4689	1314	2805	687	0.0019

^a^ One rat was removed as an outlier from the combination dosing group for HCQ.

While the 2‐DG concentration in the single dosed‐rats kept falling between 4 and 8 h, there was a modest rebound of 2‐DG by 8 h (1088 ng/mL) in the combination‐dosed rats in comparison with 4 h (464 ng/mL, *p* = .007) (Figure 3A). Since 2‐DG is phosphorylated by HKs upon uptake into the liver from the portal system and by glucose‐metabolizing organs with the end product 2‐DG‐6‐P not degradable by the glycolytic pathway and trapped intracellularly, the presence of HCQ in these tissues in the combination‐dosed rats might have altered their cellular autophagic flux and energetic state to favor an efflux of 2‐DG, possibly from the trapped 2‐DG‐6‐P, from these tissues by dephosphorylation by phosphatases.

### 3.4 HCQ PK metrics

The serum HCQ concentration versus time profiles for the HCQ post single dose or combined with 2‐DG are shown in Figure 3B. A seemingly bi‐modal time course (0.5 and 2 h) was suggested under each dosing condition (i.e., no rat peaked at 1 h). When HCQ was dosed alone (Figure 3B, open circles, solid line), *T*_max_ was on average 1.2 h (five rats with 0.5 h; four rats with 2 h, total nine rats) and average *C*_max_ was 627 ng/mL (Table 1). The combination dosing with 2‐DG (Figure 3B, open squares, dash line) delayed *T*_max_ to 2 h (all nine rats at 2 h, *p* = .017). The average *C*_max_ (394 ng/mL) was 63% of that reached from the single dosing and not statistically different (Table 1). The HCQ *AUC*_0‐8h_ and *AUC*_0‐∞_ from combination dosing were decreased by 31% and 40%, respectively (Table 1). The post‐peak *t*_1/2_ and MRT_0‐∞_ were approximately 5 folds longer than those for 2‐DG (Table 1), as expected from previous HCQ PK information from the literature^3^, ^10^, ^11^ and were not affected by the combination dosing with 2‐DG.

The apparent *V*_d_ for HCQ was an order of magnitude greater than that for 2‐DG (Table 1), as expected from prior knowledge of these drugs, and was not altered by the combination dosing with 2‐DG. However, the *CL* for HCQ was increased in the combination‐dosed rats than single dosing alone (Table 1).

The observed *C*_max_ values for HCQ were in good agreement with reported values by other authors. For example, in rats orally dosed with low, mid, and high doses of HCQ and artemisinin combination tablets, corresponding to an intake of 82, 165, and 331 mg HCQ/kg, the plasma *C*_max_ mean values were 402, 623, and 868 ng HCQ/mL, respectively.^11^ A 2023 preprint in Medrxiv (https://www.medrxiv.org/content/10.1101/2023.06.22.23291702v1) documented an oral dosing of 13.3 mg HCQ/kg to rats resulting in *C*_max_ of 90 ng HCQ/mL plasma and 183 ng HCQ/mL whole blood. In contrast, the *T*_max_ values (0.5–2 h) in our work were shorter than those reported by the other authors (4–5 h), likely influenced by the excipient vehicles for the dosing. In these reports, a bi‐modal time course pattern for blood HCQ concentration was implicated, but was not explicitly described.

## 4 DISCUSSION

### 4.1 Methodological advantages

The HPLC‐MS–MS methods reported above permit measurement of 2‐DG and HCQ in serum samples with high sensitivity, efficiency, and resolution. The small volume of bio fluid (5–10 μL serum) needed is an advantage of these sensitive methods. A running time of only 5 min for each method makes them ideal for higher‐throughput bioanalyses as well as routine PK studies of each of these drugs. It is further noteworthy that the ability of the HPLC‐MS–MS method to efficiently separate other monosaccharides permits the method to be adapted for studying these sugars with greater specificity in medicine and other fields. In terms of LLOQ/LLOD, our methods out‐performed the earlier reports by at least 1 order of magnitude.

### 4.2 Implications for precision medical oncology therapy of HK2‐addicted cancers

The PK data presented above indicated, given no change of the post‐peak *t*_1/2_ of each drug, a mutual interference of the uptake/absorption between the two drugs if orally taken simultaneously, with a more profound effect of HCQ on 2‐DG than vice versa. On reflection, the daily dosing regimen of 2‐DG in the morning and CQ in the afternoon used in our prostate cancer mouse models^1^ inadvertently avoided the brunt of a negative DDI. That is, given the mesentery‐portal absorption similarity of the i.p. injection delivery of drugs in the mouse prostate cancer models and the intragastric gavage used in the current PK study, by the time CQ was administered by i.p. injection in the afternoon, the 2‐DG that was injected in the morning had been cleared of the mesentery space to negatively impact CQ absorption. The avoidance or minimization of the interference at the mesentery level would allow CQ to effectively sabotage the autophagic survival signaling triggered by the intracellular 2‐DG and its HK2‐driven nonmetabolizable end product 2‐DG‐6‐P, in the HK2‐addicted cancer cells to drive the observed synergistic apoptosis. The rat PK interaction data warrant additional animal modeling work for optimization of dosing sequence to minimize the negative DDI in future human translation trials for therapy of HK2‐addicted castration‐resistant prostate cancer or cancers of the same metabolo‐phenotype in other organ sites.

### 4.3 Implications for other human clinical indications

Beside treating malaria and certain autoimmune diseases clinically,^4^ HCQ is approved as a third line add‐on drug for glycemic control in India for type II diabetes patients for long term use.^15^, ^16^ Although improvement of circulating insulin level and tissue insulin sensitivity have been most often cited as its putative anti‐hyperglycemia mechanisms, our observed negative PK interactions in the rat model suggest yet another and more direct mode of interaction: HCQ decreases glucose absorption (inferring from 2‐DG PK) in the gastrointestinal tract. In fact, hypoglycemia is a stated side effect of HCQ use in non‐diabetic patients (Hydroxychloroquine Tablets: Package Insert/Prescribing Information—Drugs.com). In contrast to the global failure of HCQ for COVID‐19 treatment, 2‐DG has been shown in Phase II and Phase III trials in India to improve the outcome of COVID patients as much as a median reduction of 2.5 days to achieve normalization of specific vital signs parameters when compared with standard of care.^17^

### 4.4 Speculation of how 2‐DG and HCQ affected the absorption of the other at the gastrointestinal level

Given the lack of impact of the combination dosing on post‐peak *t*_1/2_, MRT_0‐∞_ of each drug, the combination dosing of 2‐DG with HCQ might have affected the intestinal mucosal transmembrane ionic gradients/potentials or intracellular pH due to the lysosomotropic action of HCQ and the anti‐glycolytic action of 2‐DG. By analogy to glucose absorption from the intestine mucosa, such changes might have negatively impacted the efficiency of the mucosal SGLT1 and basolateral GLUT2 glucose transporters^18^ to move 2‐DG from the gut to the portal blood. Conversely, the presence of 2‐DG in the enterocytes might have altered the glycolytic metabolism flux to change the intracellular pH, which could attenuate the transport of HCQ, known to be pH sensitive,^3^ to the portal blood. The exact mechanisms await further investigation.

### 4.5 Study limitations

A number of changes could have improved the comparability of the PK metrics to those reported in the literature for these drugs and the rigor of the study. These include (1) more time points for blood collection beyond 8 h for HCQ due to its known long elimination half‐life and extreme large *V*_d_; (2) more time points immediately after 2‐DG gavage to more accurately assess its *T*_max_ and *C*_max_; (3) a validation PK experiment in which 2‐DG dosing would precede HCQ dosing for 3–5 h, mimicking the dosing delay in our mouse anti‐cancer efficacy studies,^1^ to demonstrate the minimization or avoidance of the PK interference on HCQ.

## 5 CONCLUSIONS

The cutting‐edge analytical methodologies of HPLC‐MS–MS MRM enabled the assessment of PK metrics of 2‐DG and HCQ in a rat model. The data suggest significant negative PK interactions between the two oral drugs taken simultaneously, probably at the intestinal absorption level. The data warrant additional animal modeling work for optimizing their dosing sequence to minimize the negative DDIs in future human translation trials for cancer therapy or other indications.

## AUTHOR CONTRIBUTIONS

D.S. developed HPLC‐MS–MS methods and analyzed the serum samples. S.K. and D.K. performed all animal experiments and collected and prepared blood samples. S.K. and J.L. designed the animal experiments. Y.D. and C.J. contributed to study design, resources, and data interpretation. J.L. and Y.D. conceived the project and obtained grant funding as multi‐principal investigators. D.S. and J.L. wrote the main manuscript text, prepared all figures and tables. All authors reviewed and approved the manuscript. D.S. and J.L. are corresponding authors.

## FUNDING INFORMATION

This work was funded in part by The United States National Cancer Institute, R21 CA218774 grant (to JL; YD).

## CONFLICT OF INTEREST STATEMENT

The authors have no conflicts of interest to declare.

## ETHICS STATEMENT

The animal work had been conducted with the approval of the Institutional Animal Care and Use Committee of Penn State College of Medicine, Hershey, PA campus.

## Supporting information


Appendix S1:

## Data Availability

The data that support the findings of this study are available in the supplementary material of this article.
