# Supplementary material for: 2‐Deoxyglucose and hydroxychloroquine HPLC‐MS–MS analytical methods and pharmacokinetic interactions after oral co‐administration in male rats
Source: Pharmacol Res Perspect. 2024 Jan 31;12(1):e1173. doi: 10.1002/prp2.1173 (PMC10829054; doi:10.1002/prp2.1173)
Supplement: Supplementary file 1 — Appendix S1: [file PRP2-12-e1173-s001.pptx]

## Slide 1
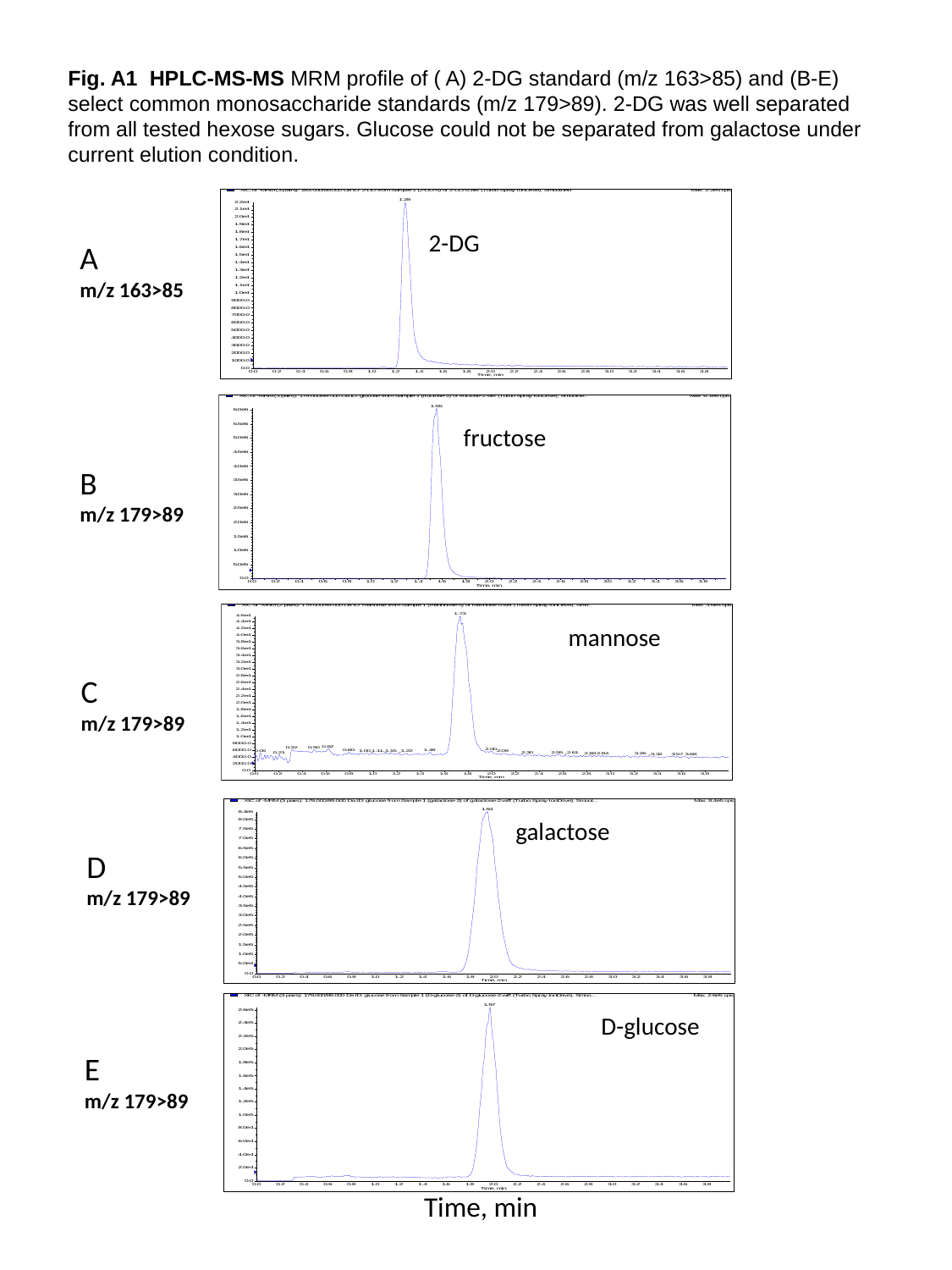

Fig. A1 HPLC-MS-MS MRM profile of ( A) 2-DG standard (m/z 163>85) and (B-E) select common monosaccharide standards (m/z 179>89). 2-DG was well separated from all tested hexose sugars. Glucose could not be separated from galactose under current elution condition.
2-DG
A
m/z 163>85
fructose
B
m/z 179>89
mannose
C
m/z 179>89
galactose
D
m/z 179>89
D-glucose
E
m/z 179>89
Time, min

## Slide 2
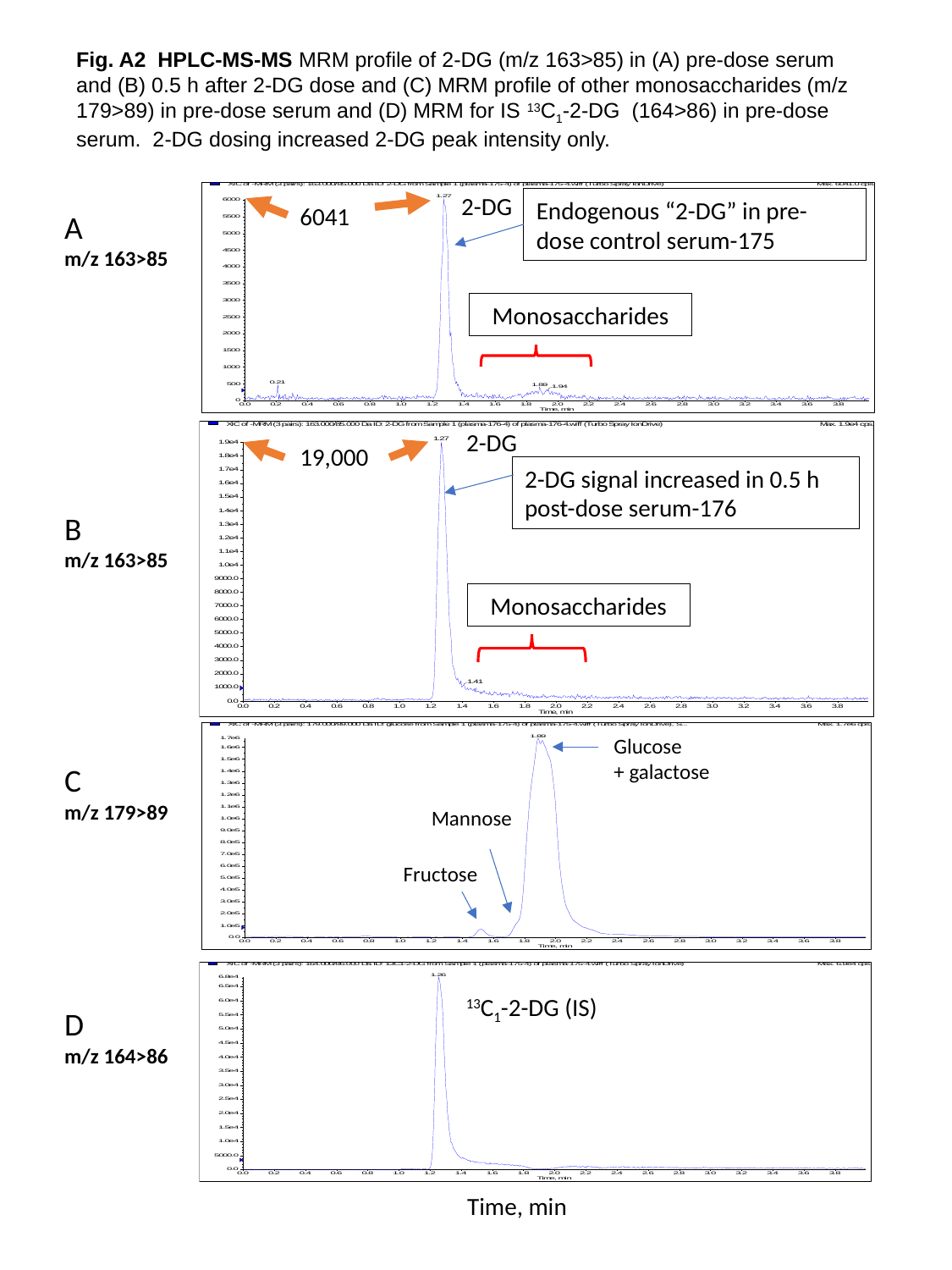

Fig. A2 HPLC-MS-MS MRM profile of 2-DG (m/z 163>85) in (A) pre-dose serum and (B) 0.5 h after 2-DG dose and (C) MRM profile of other monosaccharides (m/z 179>89) in pre-dose serum and (D) MRM for IS 13C1-2-DG (164>86) in pre-dose serum. 2-DG dosing increased 2-DG peak intensity only.
2-DG
6041
Endogenous “2-DG” in pre-dose control serum-175
Monosaccharides
A
m/z 163>85
2-DG
19,000
2-DG signal increased in 0.5 h post-dose serum-176
Monosaccharides
B
m/z 163>85
Glucose
+ galactose
Mannose
Fructose
C
m/z 179>89
13C1-2-DG (IS)
D
m/z 164>86
Time, min

## Slide 3
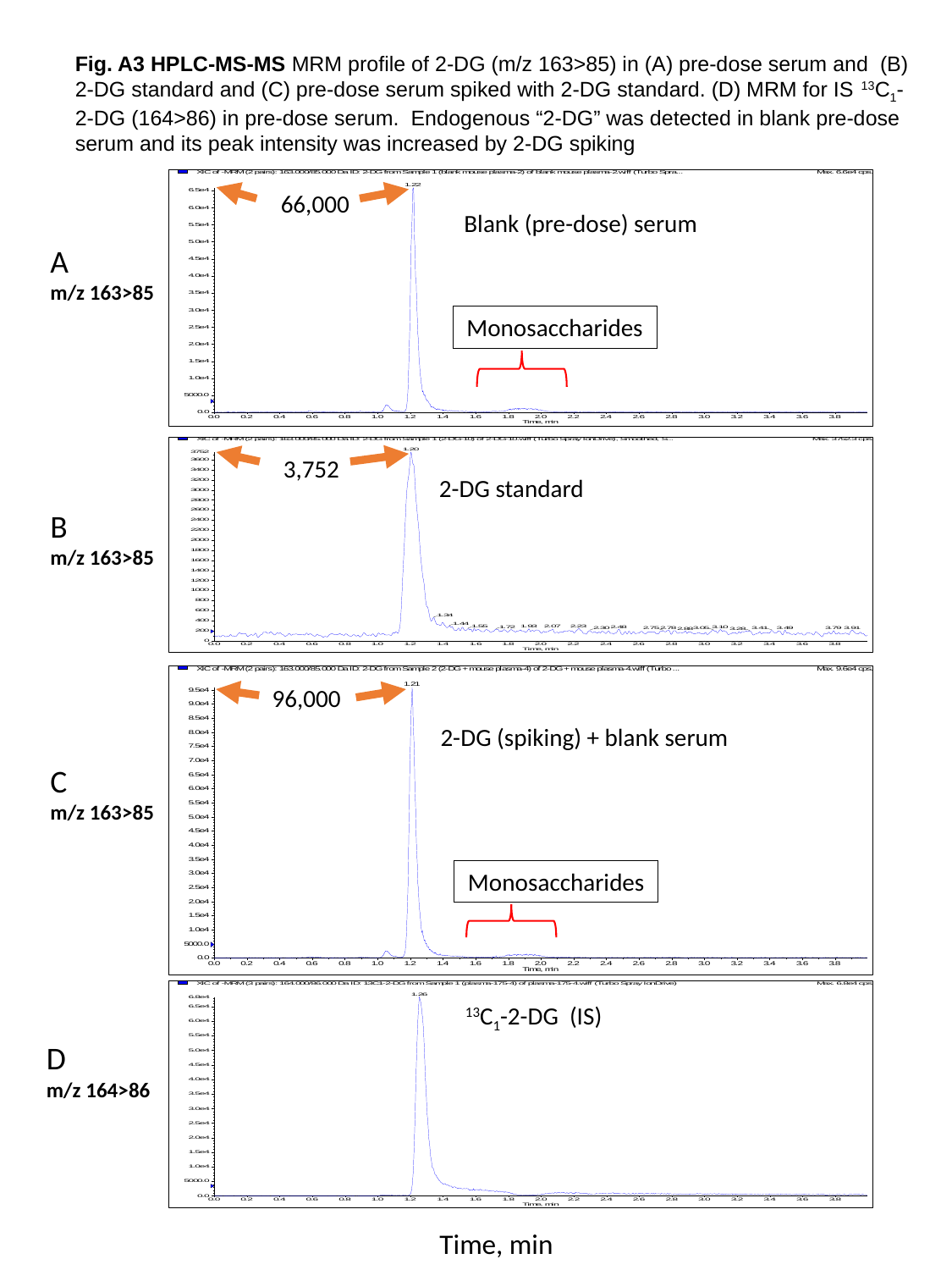

Fig. A3 HPLC-MS-MS MRM profile of 2-DG (m/z 163>85) in (A) pre-dose serum and (B) 2-DG standard and (C) pre-dose serum spiked with 2-DG standard. (D) MRM for IS 13C1-2-DG (164>86) in pre-dose serum. Endogenous “2-DG” was detected in blank pre-dose serum and its peak intensity was increased by 2-DG spiking
66,000
Blank (pre-dose) serum
Monosaccharides
A
m/z 163>85
3,752
2-DG standard
B
m/z 163>85
96,000
2-DG (spiking) + blank serum
Monosaccharides
C
m/z 163>85
13C1-2-DG (IS)
D
m/z 164>86
Time, min

## Slide 4
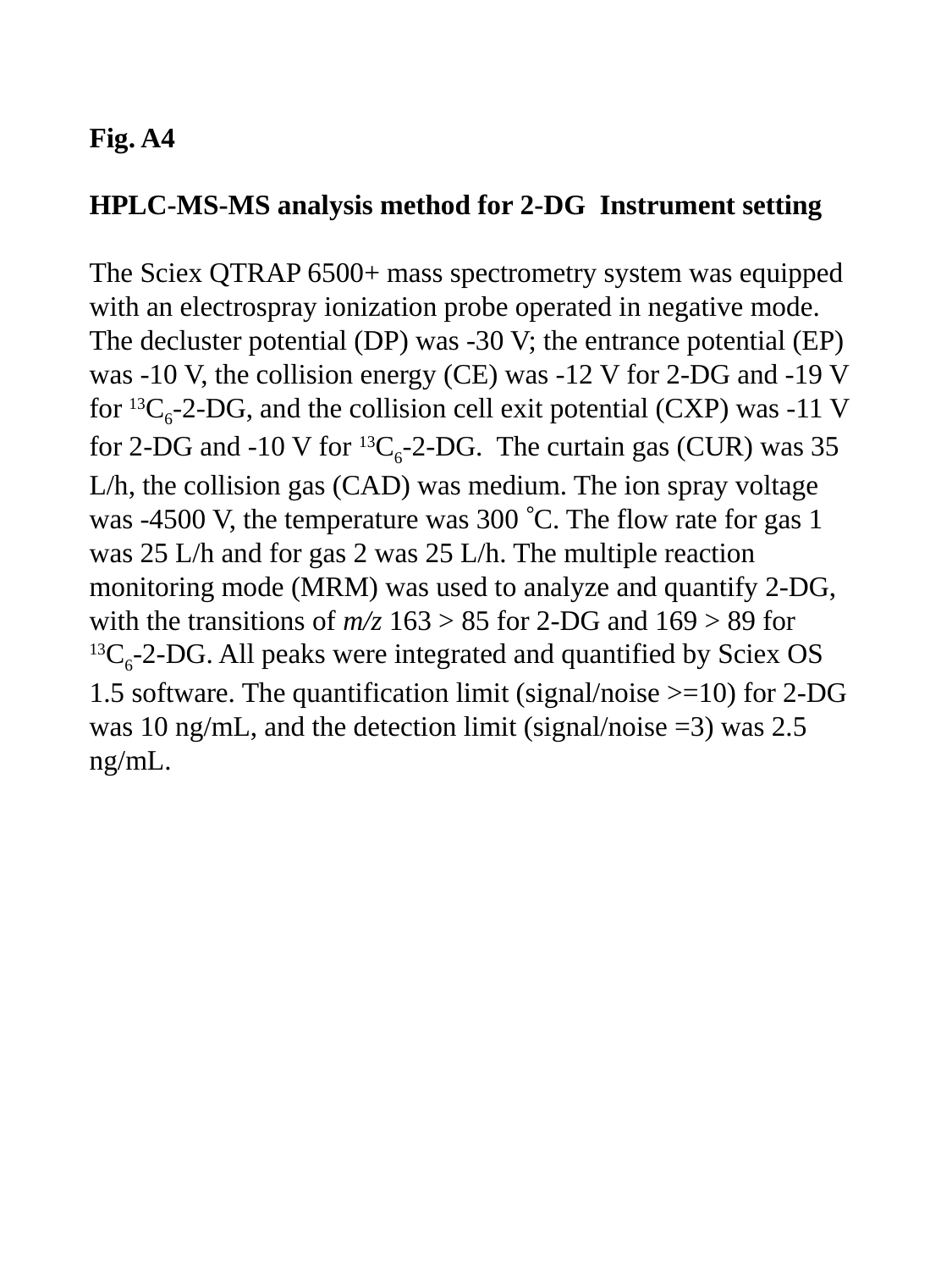

Fig. A4
HPLC-MS-MS analysis method for 2-DG Instrument setting
The Sciex QTRAP 6500+ mass spectrometry system was equipped with an electrospray ionization probe operated in negative mode. The decluster potential (DP) was -30 V; the entrance potential (EP) was -10 V, the collision energy (CE) was -12 V for 2-DG and -19 V for 13C6-2-DG, and the collision cell exit potential (CXP) was -11 V for 2-DG and -10 V for 13C6-2-DG. The curtain gas (CUR) was 35 L/h, the collision gas (CAD) was medium. The ion spray voltage was -4500 V, the temperature was 300 °C. The flow rate for gas 1 was 25 L/h and for gas 2 was 25 L/h. The multiple reaction monitoring mode (MRM) was used to analyze and quantify 2-DG, with the transitions of m/z 163 > 85 for 2-DG and 169 > 89 for 13C6-2-DG. All peaks were integrated and quantified by Sciex OS 1.5 software. The quantification limit (signal/noise >=10) for 2-DG was 10 ng/mL, and the detection limit (signal/noise =3) was 2.5 ng/mL.

## Slide 5
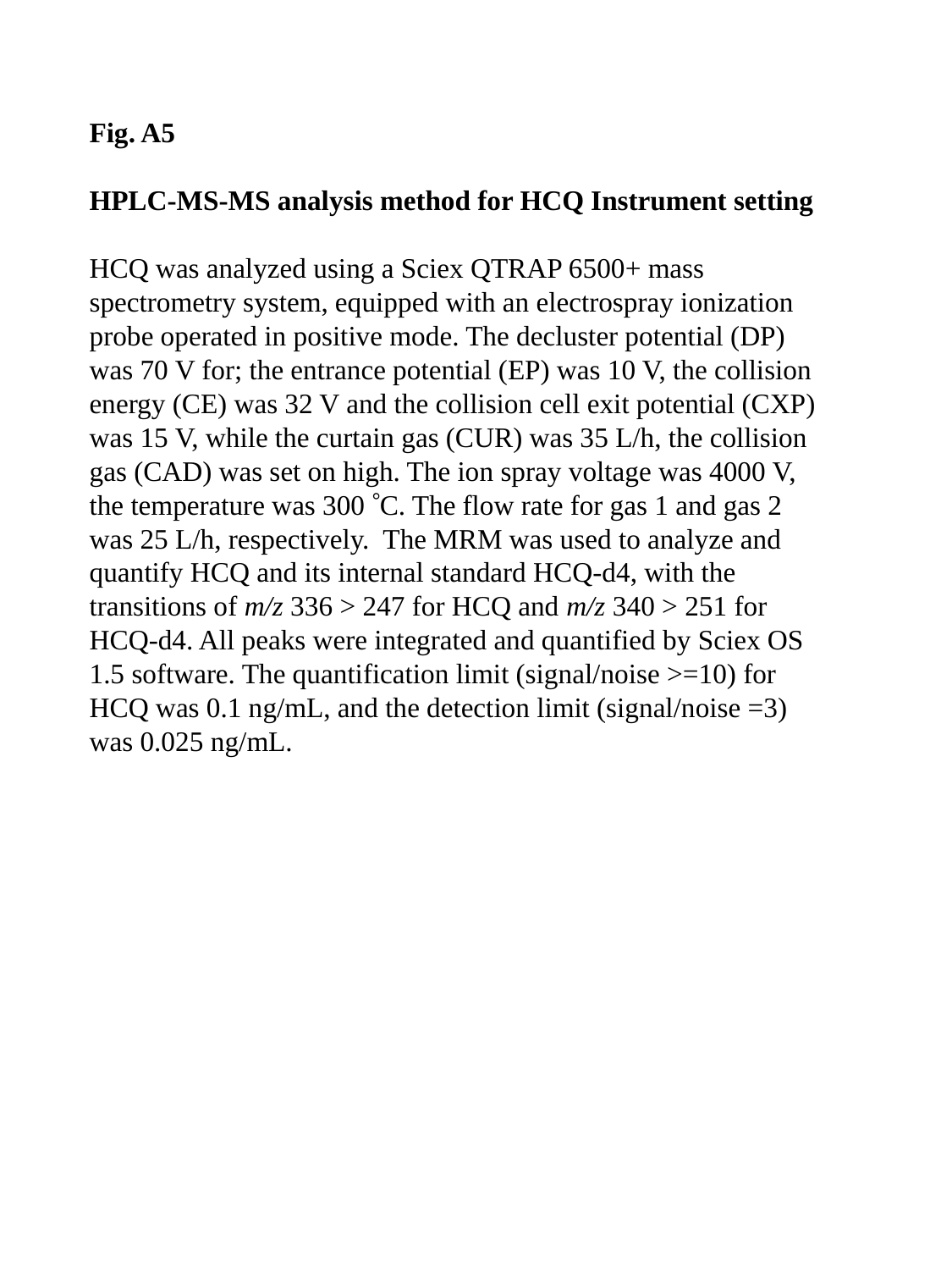

Fig. A5
HPLC-MS-MS analysis method for HCQ Instrument setting
HCQ was analyzed using a Sciex QTRAP 6500+ mass spectrometry system, equipped with an electrospray ionization probe operated in positive mode. The decluster potential (DP) was 70 V for; the entrance potential (EP) was 10 V, the collision energy (CE) was 32 V and the collision cell exit potential (CXP) was 15 V, while the curtain gas (CUR) was 35 L/h, the collision gas (CAD) was set on high. The ion spray voltage was 4000 V, the temperature was 300 °C. The flow rate for gas 1 and gas 2 was 25 L/h, respectively. The MRM was used to analyze and quantify HCQ and its internal standard HCQ-d4, with the transitions of m/z 336 > 247 for HCQ and m/z 340 > 251 for HCQ-d4. All peaks were integrated and quantified by Sciex OS 1.5 software. The quantification limit (signal/noise >=10) for HCQ was 0.1 ng/mL, and the detection limit (signal/noise =3) was 0.025 ng/mL.

## Slide 6
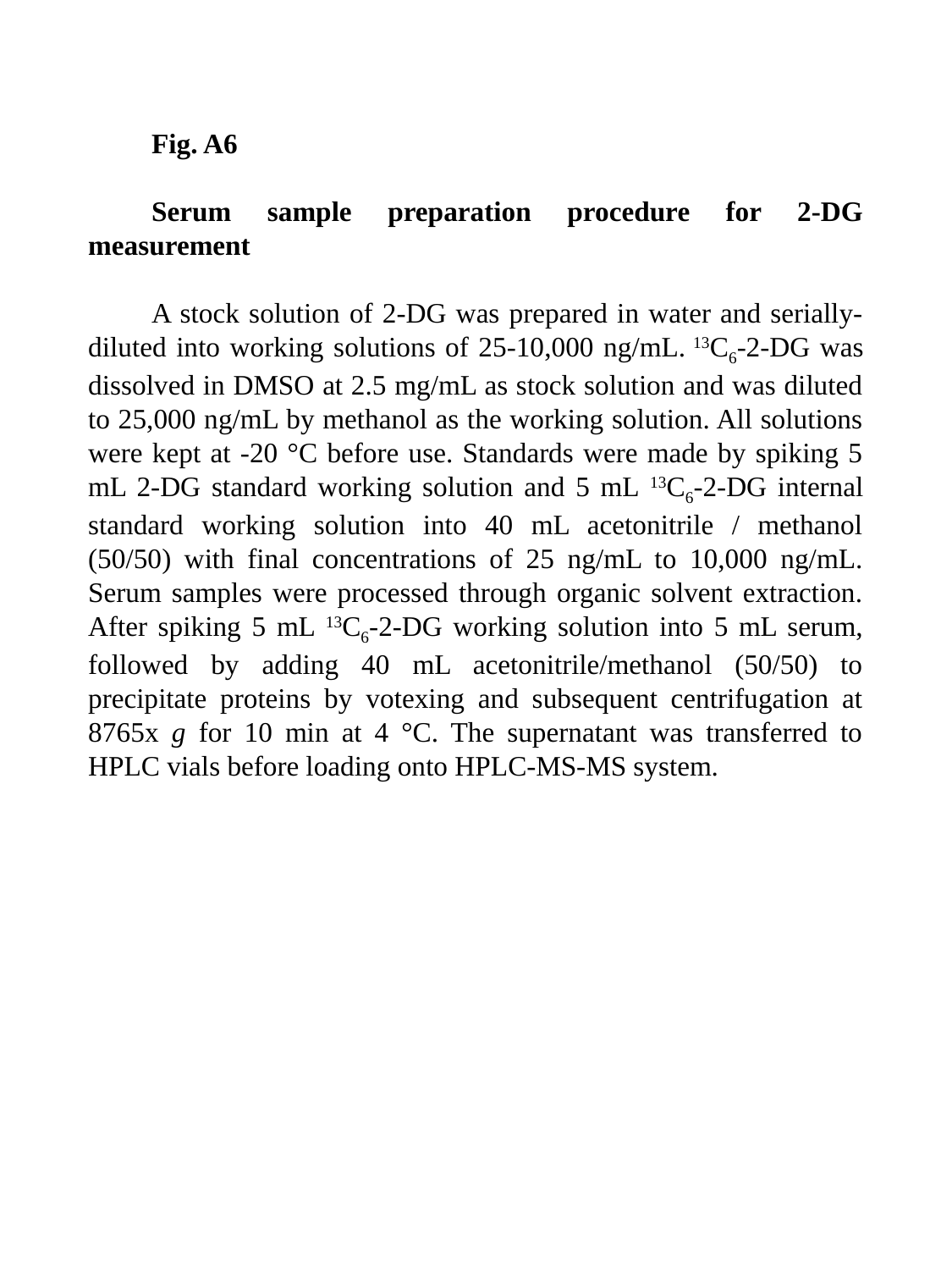

Fig. A6
Serum sample preparation procedure for 2-DG measurement
A stock solution of 2-DG was prepared in water and serially-diluted into working solutions of 25-10,000 ng/mL. 13C6-2-DG was dissolved in DMSO at 2.5 mg/mL as stock solution and was diluted to 25,000 ng/mL by methanol as the working solution. All solutions were kept at -20 °C before use. Standards were made by spiking 5 mL 2-DG standard working solution and 5 mL 13C6-2-DG internal standard working solution into 40 mL acetonitrile / methanol (50/50) with final concentrations of 25 ng/mL to 10,000 ng/mL. Serum samples were processed through organic solvent extraction. After spiking 5 mL 13C6-2-DG working solution into 5 mL serum, followed by adding 40 mL acetonitrile/methanol (50/50) to precipitate proteins by votexing and subsequent centrifugation at 8765x g for 10 min at 4 °C. The supernatant was transferred to HPLC vials before loading onto HPLC-MS-MS system.

## Slide 7
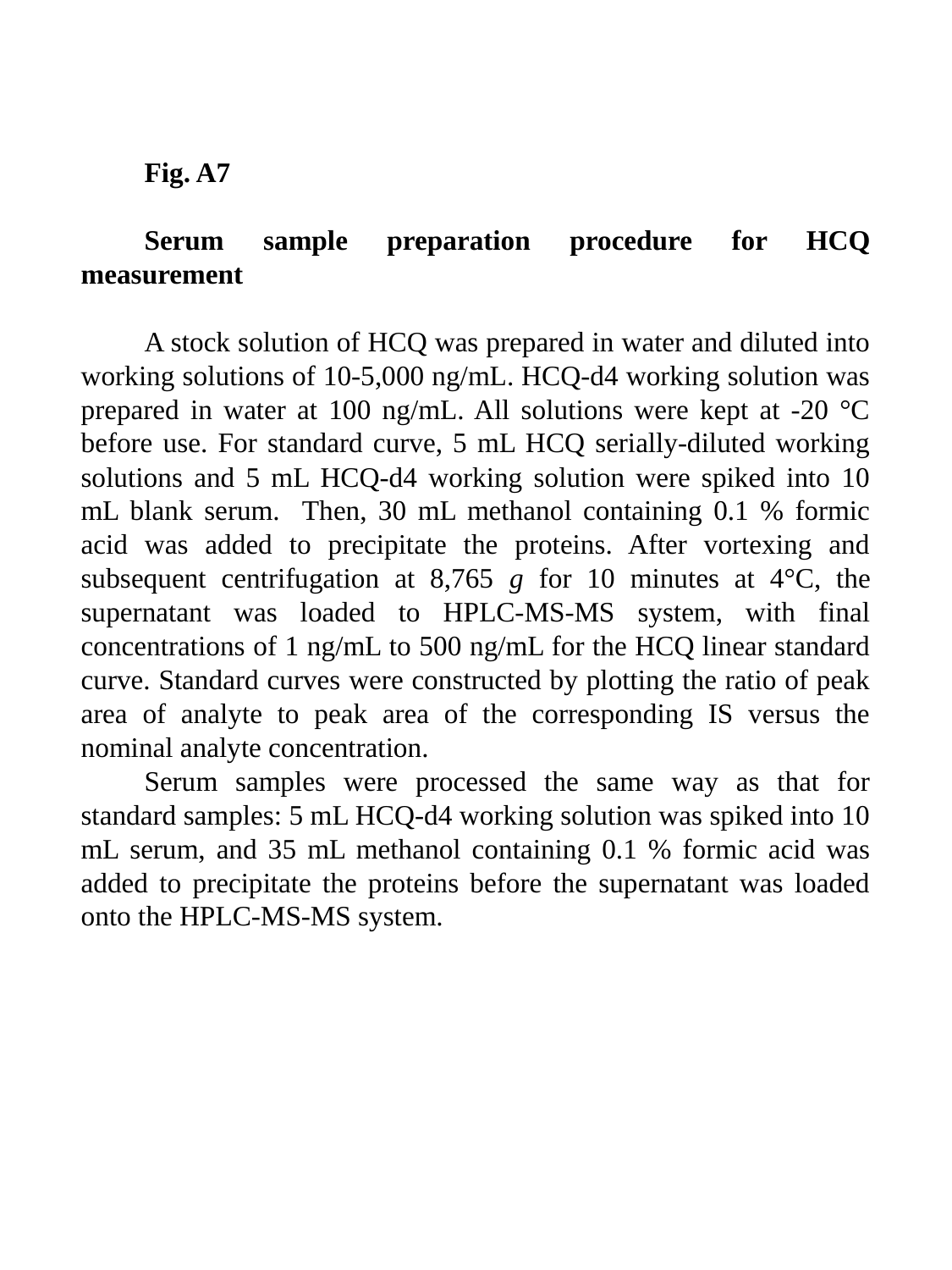

Fig. A7
Serum sample preparation procedure for HCQ measurement
A stock solution of HCQ was prepared in water and diluted into working solutions of 10-5,000 ng/mL. HCQ-d4 working solution was prepared in water at 100 ng/mL. All solutions were kept at -20 °C before use. For standard curve, 5 mL HCQ serially-diluted working solutions and 5 mL HCQ-d4 working solution were spiked into 10 mL blank serum. Then, 30 mL methanol containing 0.1 % formic acid was added to precipitate the proteins. After vortexing and subsequent centrifugation at 8,765 g for 10 minutes at 4°C, the supernatant was loaded to HPLC-MS-MS system, with final concentrations of 1 ng/mL to 500 ng/mL for the HCQ linear standard curve. Standard curves were constructed by plotting the ratio of peak area of analyte to peak area of the corresponding IS versus the nominal analyte concentration.
Serum samples were processed the same way as that for standard samples: 5 mL HCQ-d4 working solution was spiked into 10 mL serum, and 35 mL methanol containing 0.1 % formic acid was added to precipitate the proteins before the supernatant was loaded onto the HPLC-MS-MS system.

## Slide 8
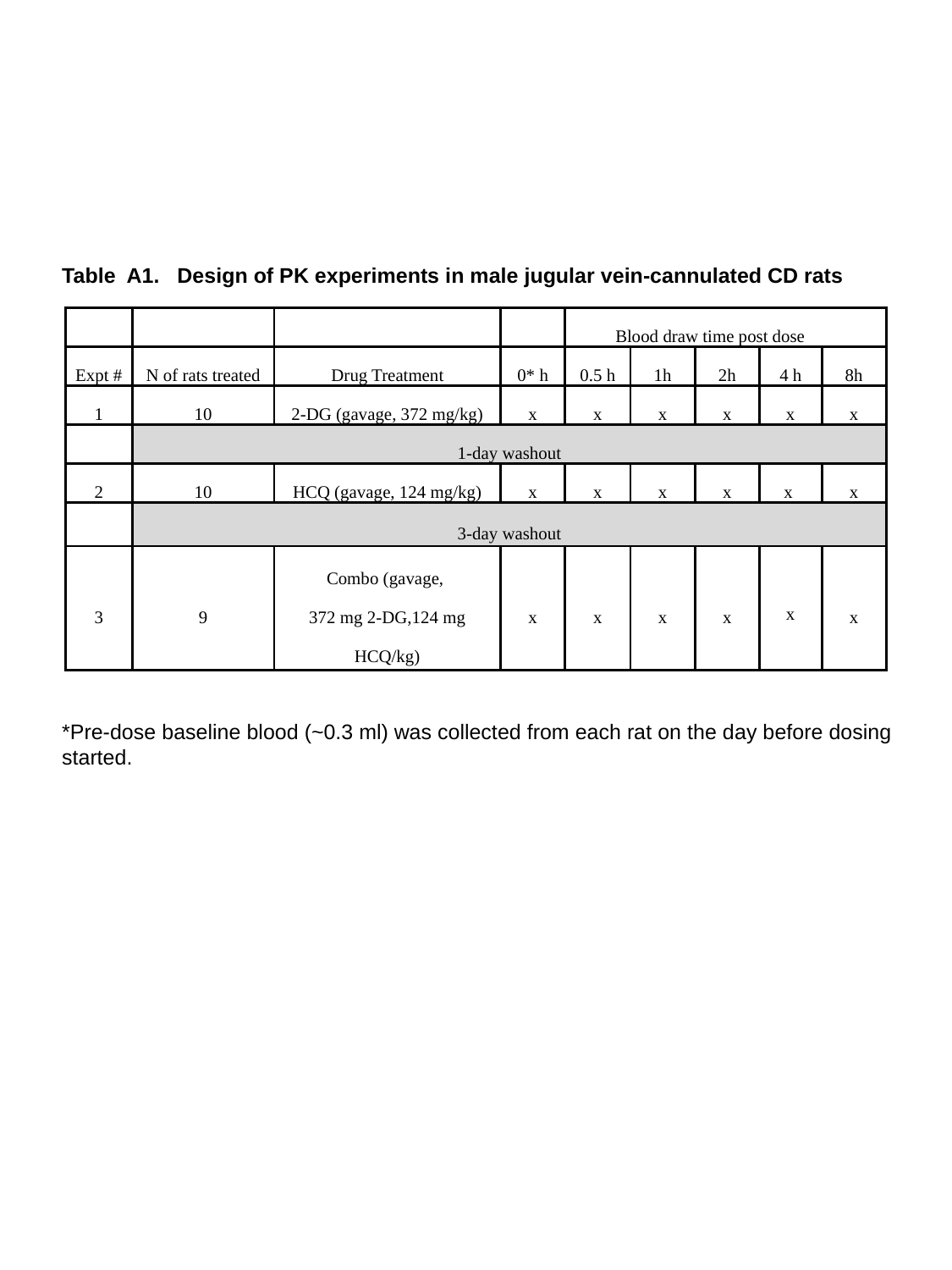

Table A1. Design of PK experiments in male jugular vein-cannulated CD rats
 . .
*Pre-dose baseline blood (~0.3 ml) was collected from each rat on the day before dosing started.
| | | | | Blood draw time post dose | | | | |
| --- | --- | --- | --- | --- | --- | --- | --- | --- |
| Expt # | N of rats treated | Drug Treatment | 0\* h | 0.5 h | 1h | 2h | 4 h | 8h |
| 1 | 10 | 2-DG (gavage, 372 mg/kg) | x | x | x | x | x | x |
| | 1-day washout | | | | | | | |
| 2 | 10 | HCQ (gavage, 124 mg/kg) | x | x | x | x | x | x |
| | 3-day washout | | | | | | | |
| 3 | 9 | Combo (gavage, 372 mg 2-DG,124 mg HCQ/kg) | x | x | x | x | x | x |
